# Computed tomography findings of thyroid hemiagenesis: differentiation from hemithyroidectomy

**DOI:** 10.1186/s12880-023-00961-3

**Published:** 2023-01-10

**Authors:** Dae Young Yoon, Eun Soo Kim, Chae Woon Lee, Young Lan Seo, Yul Lee, Mi Jung Kwon, Sang Min Lee

**Affiliations:** 1 Department of Radiology, Kangdong Sacred Heart Hospital, Hallym University College of Medicine, Seoul, Republic of Korea; 2 Department of Radiology, Hallym University Sacred Heart Hospital, Hallym University College of Medicine, 22, Gwanpyeong-ro 170beon-gil, Dongan-gu, Anyang-si, Gyeonggi-do 14068 Republic of Korea; 3 Department of Pathology, Hallym University Sacred Heart Hospital, Hallym University College of Medicine, Anyang-si, Republic of Korea; 4 Department of Radiology, Gangnam Medical Center, Cha University, Seoul, Republic of Korea

**Keywords:** Thyroid hemiagenesis, Hemithyroidectomy, CT, Differential diagnosis

## Abstract

**Objectives:**

Thyroid hemiagenesis is a rare congenital anomaly characterized by the lack of development of one thyroid lobe. The purpose of this study was to evaluate computed tomography (CT) findings of thyroid hemiagenesis and to establish useful CT criteria for differentiating thyroid hemiagenesis from the hemithyroidectomy state.

**Methods:**

The CT images of 11 patients with thyroid hemiagenesis were retrospectively reviewed and compared with those of 100 (49 left and 51 right) patients in a hemithyroidectomy state. Image analysis was performed according to the following CT parameters: (a) side of thyroid hemiagenesis, (b) edge of the medial end of the remnant thyroid gland, (c) location of the medial end of the remnant thyroid gland, expressed as the angle of the medial end and (d) any other thyroid abnormality observed during the initial examination.

**Results:**

The missing lobe occurred more often in the left than in the right lobe (72.7% vs. 27.3%) as well as concomitant isthmus agenesis (100% vs. 37.5%). The sharp edge of the medial end of the remnant thyroid gland was more common in thyroid hemiagenesis (64%) than in hemithyroidectomy (26%) (*P* = 0.0153). In left thyroid hemiagenesis, the angle of the medial end (63%) was more frequently >  + 30° than in hemithyroidectomy (0%) (*P* < 0.0001). Two patients presented with hypothyroidism; the remaining nine showed a normal thyroid function. The associated thyroid diseases were autoimmune thyroiditis (n = 1) and papillary thyroid carcinoma (n = 1).

**Conclusions:**

The sharp edge of the medial end of the remnant thyroid gland and an angle of >  + 30° for the medial end in cases wherein the left lobe is absent are useful CT features for distinguishing thyroid hemiagenesis from hemithyroidectomy.

## Introduction

Thyroid hemiagenesis is a rare congenital anomaly in which one lobe (and sometimes the isthmus,as well) of the thyroid gland fails to develop [1]. Its prevalence was reported to be 0.02–0.2% in the normal population [2–5].

Although the exact pathogenesis of thyroid hemiagenesis is unknown, some environmental and genetic alterations in the transcriptional control of thyroid development have been suggested [6, 7]. Given that the absence of one thyroid lobe usually causes no symptoms or clinical consequences by itself [7], the presence of this anomaly is detected incidentally in clinical setting while investigating concomitant pathologies of the remaining thyroid lobe or the neck region [1, 6, 7].

To date, a few previous reports described the imaging findings of thyroid hemiagenesis as absence of the one thyroid lobe in scintigraphy, ultrasonography (US), and computed tomography (CT) [7–10]. However, absence of one thyroid lobe in imaging study may strongly suggest the diagnosis of hemithyroidectomy state in patients for whom no or little clinical information is available. To the best of our knowledge, there has been no comprehensive analysis of the imaging findings of thyroid hemiagenesis.

The purpose of this study was to evaluate CT findings of thyroid hemiagenesis and to establish useful CT criteria for differentiating thyroid hemiagenesis from the hemithyroidectomy state.

## Materials and methods

This study conformed to the principles of the Helsinki Declaration of 1975 (revised version 2013). The study protocol was approved by the Institutional Review Board of Hallym University Sacred Heart hospital (No. 2021-12-006-001), which waived the need for written informed consent from the subjects due to the retrospective nature of the study.

### Subjects

We conducted a cross-search of the medical record database and radiologic information system for patients with thyroid hemiagenesis. Thyroid hemiagenesis was defined as the congenital total absence of one thyroid lobe on CT and/or US. Between January 2006 and October 2021, we identified 11 patients in whom the final diagnosis was thyroid hemiagenesis. For each patient with thyroid hemiagenesis, we retrieved and recorded the following clinical data at the time of the initial evaluation of the patient: sex, age, cause of referral, thyroid function test, family history, and associated thyroid disorder. Thyroid function was studied by measuring thyroid hormones (free thyroxine [T4], free triiodothyronine [T3], and thyroid-stimulating hormone [TSH]) and thyroid antibodies (antithyroid peroxidase and antithyroglobulin). Hypothyroidism was defined as TSH level above the reference values, while hyperthyroidism was diagnosed when TSH concentration was below the normal level.

For comparison with the thyroid hemiagenesis group, we identified 100 patients, matched for age and gender, who underwent right (n = 51) and left (n = 49) hemithyroidectomy and follow-up CT of the neck between January 2020 and October 2021.

### CT examination

Contrast-enhanced neck CT examinations were performed with a 16-row (MX8000 Infinite Detector Technology; Philips Medical Systems) or a 256-row multidetector CT scanner (Brilliance iCT, Philips Medical Systems) or a 128-row multidetector CT scanner (Somatom Definition Flash 256, Siemens Healthineers) or a 128-edge CT (Siemens Healthineers). The technical parameters were as follows: pitch of 1.5 or 0.61; gantry rotation time of 50 ms; collimation of 4 × 1.5 mm or 6.4 × 0.625 mm; 512 × 512 matrix; 120 kVp; and 200 mAs. Image acquisition was started 90 s after intravenous injection (80–100 mL) of iohexol (300 mg/mL, Omnipaque 300, GE Healthcare) or iomeprol (350 mg/mL, Iomeron 350, Bracco) at a rate of 2–3 mL/s administered with an automatic power injector. Axial images were reconstructed with contiguous slices of 3-mm thickness.

### Analysis

Two radiologists (D.Y.Y. and E.S.K with 25 and 15 years of thyroid imaging experience, respectively) blinded to the proved diagnosis and results of other reviewers, reviewed the CT findings retrospectively. Any disagreements between the two reviewers were resolved by consecutive consensus reading.

**Fig. 1:**
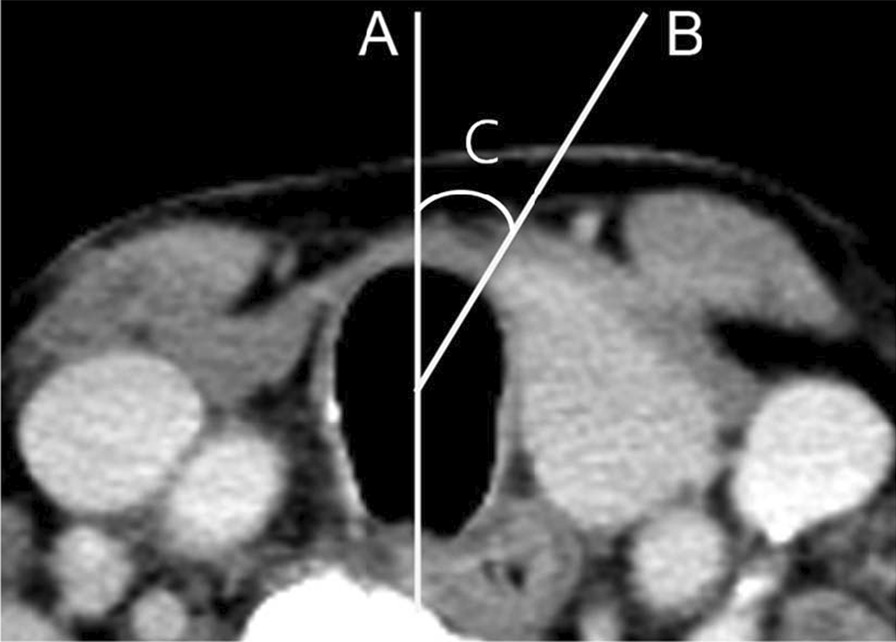
The method of measurement for the angle of the medial end of remnant thyroid gland. The location of the medial end of remnant thyroid gland is expressed as the angle of the medial end (**C**), which is defined as the angle between a vertical line drawn on the anterior tip of the trachea (**A**) and a line connecting the center of trachea to medial end of remnant thyroid gland (**B**)

To compare the CT findings of thyroid hemiagenesis and hemithyroidectomy state, the following imaging features were retrospectively analyzed: (a) side of thyroid hemiagenesis (right or left), (b) edge of the medial end of the remnant thyroid gland (sharp edge or blunt edge), (c) location of the medial end of the remnant thyroid gland, expressed as the angle of the medial end (the angle between a vertical line drawn on the anterior tip of the trachea and a line connecting the center of trachea to medial end of the remnant thyroid gland in intervals of 1°) (Fig. 1), and (d) any other thyroid abnormality observed during the initial examination.

A sharp edge was defined as an edge sharper than 30° and smaller than 2 mm in tip thickness, and a blunt edge was defined as an edge ≥ 30° or ≥ 2 mm in tip thickness. The angle of the medial end of the remnant thyroid gland was computed clockwise from 0° to + 180° and counterclockwise from 0° to –180°, with 0° being at the midline anterior tracheal wall. All measurements were performed on the workstation with an internal electronic caliper after appropriate magnification and determined by the mean value of measurements by two reviewers.

### Statistical analysis

Statistical analyses were performed using Fisher's exact test for categorical variables and Mann–Whitney U test for continuous variables to compare patients’ demographics and imaging findings between thyroid hemiagenesis and hemithyroidectomy state. A *P*-value of < 0.05 was considered to indicate a significant difference. All statistical analyses were performed using SPSS version 21.0 (IBM).

## Results

In 11 patients with thyroid hemiagenesis, seven were women, and four were men (female:male ratio, 1.75:1.00), with a mean age of 52.4 ± 14.4 years (range, 25–71 years). The patients were referred for neck CT for various reasons. In all patients, thyroid hemiagenesis was detected incidentally during the evaluation of thyroid or non-thyroidal disorders. All were unrelated individuals based on a detailed family history with regards to congenital thyroid disease.

The missing lobe was the left lobe in eight patients and the right lobe in three patients (left to right ratio, 2.67: 1.00). The isthmus was visualized in five (62.5%) of eight patients with left hemiagenesis and absent in all three patients with right hemiagenesis. Two patients presented with hypothyroidism; the remaining nine showed a normal thyroid function. The associated thyroid diseases were autoimmune thyroiditis (n = 1) and papillary thyroid carcinoma (n = 1) (Table 1).

**Table 1:** Characteristics of 11 cases with thyroid hemiagenesis

Case no	Age/sex	Cause of referral	Side of thyroid hemiagenesis	Thyroid function test	Associated thyroid disorder
1	42/F	Parotid cancer	Left	Normal	None
2	25/F	Lymphadenitis	Left	Normal	None
3	71/M	Neck pain	Left	Normal	None
4	64/M	Colon cancer metastasis	Left	Normal	None
5	65/M	Neck lipoma	Right	Normal	None
6	49/F	Regular check-up	Right	Normal	None
7	52/F	Regular check-up	Left	Normal	None
8	39/F	Intraductal papilloma in the breast	Left	Normal	None
9	46/F	Choriocarcinoma	Right	Normal	Autoimmune thyroiditis
10	52/F	Thyroid cancer	Left	Hypothyroidism	Thyroid cancer
11	71/M	Mediastinal mass	Left	Normal	None

The patient demographics and CT findings of thyroid hemiagenesis and hemithyroidectomy state are summarized in Table 2. There were no significant differences between the two groups in terms of sex (*P* = 0.109) and age (*P* = 0.946).

**Table 2:** Comparison between thyroid hemiagenesis and hemithyroidectomy state

Demographics and CT findings	Thyroid hemiagenesis (n = 11)	Hemithyroidectomy state (n = 100)	*P*-value
Sex			0.1089
Male	4 (36.4%)	16 (16%)	
Female	7 (63.6%)	84 (84%)	
Age (years)			0.9464
Mean ± standard deviation	52.4 ± 14.4	52.9 ± 13.7	
Range	25–71	25–92	
Edge of medial end			0.0153
Blunt edge	4 (36.4%)	74 (74%)	
Sharp edge	7 (63.6%)	26 (26%)	
Location of right medial end (absence of left lobe)	8	49	P < 0.0001
< − 61°	0	2 (4.1%)	
− 31° to − 60°	0	8 (16.3%)	
− 30° to + 30°	3 (37.5%)	39 (79.6%)	
+ 31° to + 60°	4 (50.0%)	0	
> + 61°	1 (12.5%)	0	
Location of left medial end (absence of right lobe)	3	51	0.6631
< − 61°	0	0	
− 31° to− 60°	0	0	
− 30° to + 30°	3 (100%)	21 (41.2%)	
+ 31° to + 60°	0	24 (47.0%)	
> + 61°	0	6 (11.8%)	

**Fig. 2:**
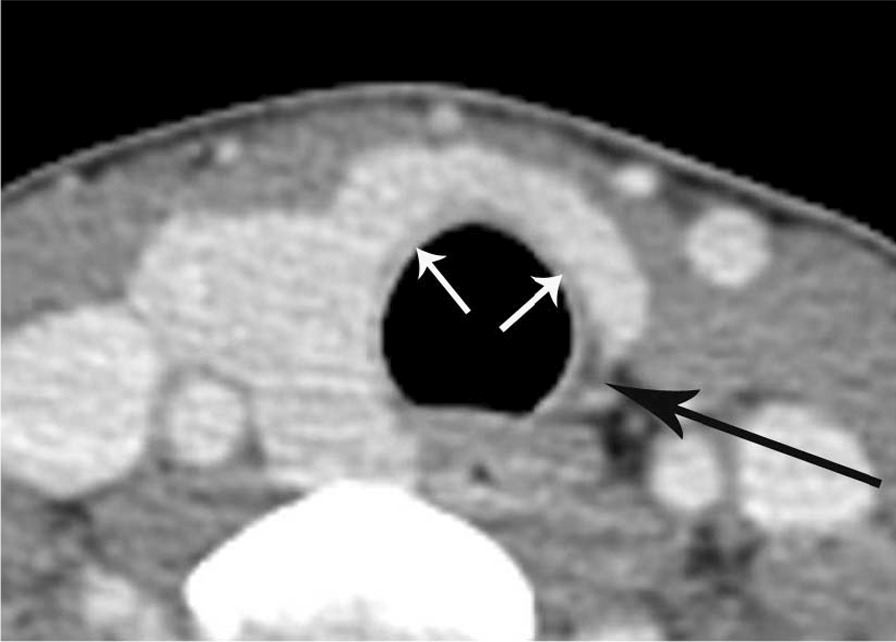
A 42-year-old woman with left thyroid hemiagenesis (case 1). Axial contrast-enhanced CT scan shows the presence of isthmus (short white arrows) and sharp edge of medial end of remnant thyroid gland (arrow). The angle of the medial end of remnant thyroid gland was measured as + 108°

**Fig. 3:**
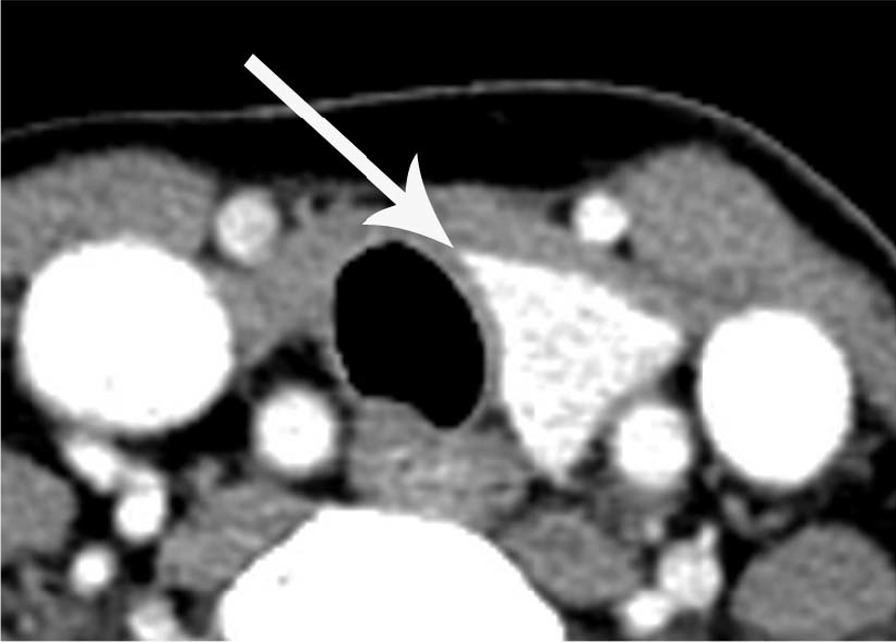
A 65-year-old woman with right thyroid hemiagenesis (case 5). Axial contrast-enhanced CT scan shows the absence of isthmus and sharp edge of medial end of remnant thyroid gland (arrow). The angle of the medial end of remnant thyroid gland was measured as + 29°

**Fig. 4:**
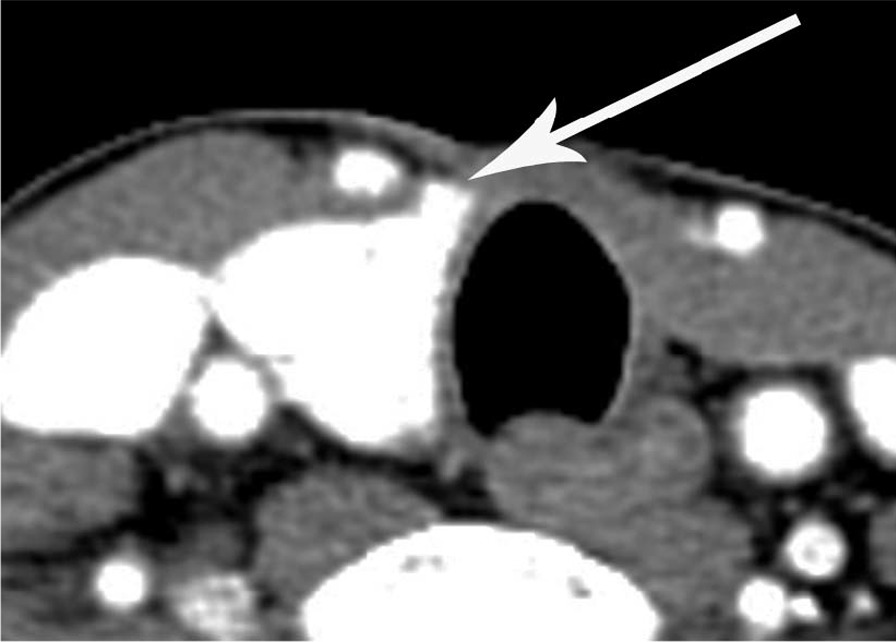
A 61-year-old woman with left hemithyroidectomy for papillary thyroid cancer. Axial contrast-enhanced CT scan shows the absence of isthmus and blunt edge of medial end of remnant thyroid gland (arrow). The angle of the medial end of remnant thyroid gland was measured as − 36°

**Fig. 5:**
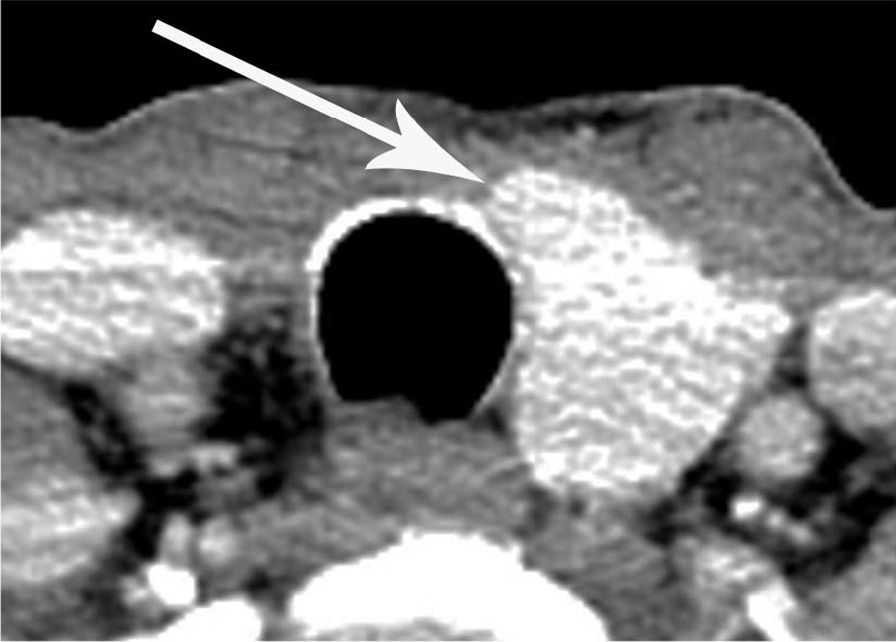
A 65-year-old man with right hemithyroidectomy for papillary thyroid cancer. Axial contrast-enhanced CT scan shows the absence of isthmus and blunt edge of medial end of remnant thyroid gland (arrow). The angle of the medial end of remnant thyroid gland was measured as + 24°

The sharp edge of the medial end of the remnant thyroid gland was found in seven cases (63.6%) of thyroid hemiagenesis (Figs. 2 and 3) and in 26 cases (26.0%) of hemithyroidectomy state, which was statistically significant between the two groups (*P* = 0.015). The other cases in each group had a blunt edge for the medial end of the remnant thyroid gland (Figs. 4 and 5). The angle of the medial end of the remnant thyroid gland was ≥ 31° in five (62.5%) of eight patients with left thyroid hemiagenesis (Fig. 2), whereas it was ≤ 30° in all 49 patients with left hemithyroidectomy state (Fig. 4) (*P* < 0.0001). The angle of the medial end of the remnant thyroid gland was between − 30° and + 30° in all three patients with right thyroid hemiagenesis (Fig. 3) and variable within the range of − 60° to + 60° (Fig. 5) in patients with right hemithyroidectomy state (*P* = 0.6631).

## Discussion

The thyroid gland is the earliest developing endocrine organ during embryogenesis. Normal thyroid gland development starts with a tubular invagination of the primitive pharyngeal endoderm at the foramen cecum, which then elongates and forms a bilobed diverticulum. This thyroid primordium descends to the lower neck along the thyroglossal duct line, gradually expands laterally, and leads to the formation of the final bilobed structure with a distinct isthmus [11, 12]. There are various developmental anomalies of the thyroid gland, including aplasia, hypoplasia, ectopia, accessory thyroid tissue, agenesis of isthmus, and hemiagenesis. Thyroid hemiagenesis is a rare congenital anomaly of the thyroid gland with absence of a lobe or a lobe and the isthmus [13].

The exact mechanisms responsible for thyroid hemiagenesis remain to be clarified. Suggested mechanisms include a defect in the descent or lobulation process of the thyroid anlage that may lead to the development of only one thyroid lobe. It is unclear whether this developmental disturbance is due to the interference of environmental factors or some genetic abnormality [12].

Most previous studies based on observations in patients affected by thyroid diseases [1, 9, 14–16] reported that thyroid hemiagenesis is commonly associated with various thyroid disorder and is more frequently found in female than in male patients. However, large surveys in a normal population [2–5] have claimed that the high incidence of thyroid abnormalities and female preponderance reported by previous studies could be related to selection bias in referring patients for thyroid evaluation. In normal population studies, the remnant thyroid lobe was usually normal, most patients were clinically euthyroid, and the female-to-male ratio was almost equal in the subjects with thyroid hemiagenesis. In our series, two of 11 patients had associated thyroid disorder, and one had hypothyroidism, with a female-to-male ratio of 1.75:1.

According to previous studies focusing on US or scintigraphic findings of thyroid hemiagenesis, involvement of the left lobe was abundantly more frequent, being present in approximately 80% of cases [1–3, 9, 14, 15, 17]. Moreover, in most previous studies, right lobe hemiagenesis was predominantly associated with isthmus agenesis. On the other hand, the isthmus was present in most cases of left lobe hemiagenesis [9, 14, 17]. Although the exact cause of these differences between right and left thyroid hemiagenesis is unknown, our results showed that the missing lobe occurred more often in the left than in the right lobe (72.7% vs. 27.3%), and concomitant isthmus agenesis occurred more often in the right than in the left lobe hemiagenesis (100% vs. 37.5%), similar to data from previous studies [1–3, 9, 14, 15, 17].

Thyroid hemiagenesis and hemithyroidectomy state share many similarities in imaging findings, which may result in misinterpretation. The differentiation between these two entities, which both present as only one thyroid lobe, is usually done clinically because they can be easily differentiated based on the past medical history of the patient. In daily practice, however, reliable history cannot always be obtained from patients. In addition, the imaging characteristics of thyroid hemiagenesis can help to understand the pathogenesis of this rare congenital disease as well as other thyroid dysgenesis.

In our CT study, we emphasize the shape and location of the medial end of the remnant thyroid gland in the differentiation of thyroid hemiagenesis from the hemithyroidectomy state. According to the results of our study, the sharp edge of the medial end of the remnant thyroid gland and angle of the medial end, which is >  + 30°, in cases of the absence of left lobe are significant CT features for distinguishing thyroid hemiagenesis from hemithyroidectomy.

Although the underlying mechanism involved is uncertain, as the histopathologic correlations could not be obtained in our study, it seems likely that the sharp edge of the medial end of the remnant thyroid gland in thyroid hemiagenesis is caused by co-existing hypoplasia of the isthmus or the medial portion of the thyroid lobe. In contrast, the blunt edge of the medial end in hemithyroidectomy can be explained as the bulging of thyroid tissue through a defect in the capsule at the cutting surface. However, our data showed that an overlap exists between the shape of edge of the medial end of the remnant thyroid of thyroid hemiagenesis and hemithyroidectomy, i.e., approximately one-fourth of hemithyroidectomy patients were found to have a sharp edge, similar to that in thyroid hemiagenesis. We can speculate that underlying agenesis or hypoplasia of the isthmus could result in the sharp edge in patients with hemithyroidectomy. A recent study based on multi-detector CT reported that the prevalence of thyroid isthmus agenesis was 4.77%, and the majority of which are the tapering-edge type [18].

In our study, the isthmus was present in five patients with left thyroid hemiagenesis. In all these cases, the angle of the medial end of the remnant thyroid gland was ≥  + 31°, i.e., the isthmus was present in the left anterolateral wall as well as anterior wall of the trachea. On the other hand, the location of the medial end of the remnant thyroid tissue in patients with hemithyroidectomy should depend on the previous surgery. Hemithyroidectomy (thyroid lobectomy with isthmusectomy) is the most common surgery necessary to treat small thyroid cancer in the absence of extrathyroidal extension and cervical nodal metastases [19]. In this surgery, one thyroid lobe and isthmus is dissected,and the isthmus is cut in close proximity to the contralateral lobe [20]. All of our patients with left hemithyroidectomy state did not show any presence of the isthmus in close proximity to the ipsilateral lobe. We, therefore, propose a criterion of 'the angle of the medial end of the remnant thyroid gland was ≥  + 31°’ as a finding suggestive of left thyroid hemiagenesis. However, this criterion seems difficult to apply in right thyroid hemiagenesis because the isthmus is usually absent in right hemiagenesis.

Ultrasonography has been considered by some as the imaging modality of choice to diagnose thyroid hemiagenesis because of its wide availability, noninvasiveness, and low cost [1–5, 9, 15, 17]. Ultrasonography is also useful to detect any morphological abnormalities within the remaining lobe [1, 9, 15]. However, US is a rather subjective and operator-dependent diagnostic method. Moreover, US may be not reliable in the evaluation of the isthmus or the medial end of the remnant thyroid tissue due to probe compression during examination. The isthmus of the thyroid gland is located between the skin and the trachea and can be efficiently compressed against underlying anatomic structures by external forces [21].

Scintigraphy with ^99m^Tc-pertechnetate or ^123^I has been used in the diagnosis of thyroid hemiagenesis. Scintigraphy is helpful to diagnose functional status of remnant thyroid gland and to identify possible ectopic thyroid tissue not seen by ultrasound. However, the diagnosis of thyroid hemiagenesis based on scintigraphy could be misleading because neoplasm, contralateral autonomous solitary thyroid nodule, thyroiditis, and infiltrative diseases also result in non-visualization of one thyroid lobe [14, 17, 22].

Computed tomography can allow a more objective evaluation of the isthmus and deep portion of lobes of thyroid gland than US. In the present study, CT was able to provide sufficient information to evaluate the features of present thyroid lobe and isthmus and to make the differential diagnosis of thyroid hemiagenesis from hemithyroidectomy state.

There are several limitations to the present study. First, the number of patients enrolled was too small to analyze the relevant CT features with sufficient statistical power. Therefore, further studies in a large number of patients with various subtypes of thyroid hemiagenesis are required to confirm our results. Second, given that the patients with thyroid hemiagenesis were retrospectively collected over a 20-year period, the CT imaging parameters varied. We were unable to obtain coronal and sagittal CT images in all patients, thus evaluation was only done through the axial CT images in our study, limiting our ability to analyze detailed morphology of the remnant thyroid gland.

In conclusion, the sharp edge of the medial end of the remnant thyroid gland and an angle of >  + 30° for the medial end in cases of the absence of the left lobe are useful CT features for distinguishing thyroid hemiagenesis from hemithyroidectomy.

## Data Availability

The datasets generated and/or analysed during the current study are not publicly available due to local ownership of the data but are available from the corresponding author on reasonable request.

## Abbreviations

CT: Computed tomography
US: Ultrasonography
